# Factors Influencing Team Performance: What Can Support Teams in High-Performance Sport Learn from Other Industries? A Systematic Scoping Review

**DOI:** 10.1186/s40798-021-00406-7

**Published:** 2022-02-22

**Authors:** Benjamin Salcinovic, Michael Drew, Paul Dijkstra, Gordon Waddington, Benjamin G. Serpell

**Affiliations:** 1 Research Institute for Sport and Exercise, University of Canberra, Canberra, Australia; 2 Rehabilitation Department, Aspetar Orthopedic and Sports Medicine Hospital, Doha, Qatar; 3 Athlete Availability, Applied Technology and Innovation, Australian Institute of Sport, Bruce, Australia; 4 Department of Medical Education, Aspetar Orthopedic and Sports Medicine Hospital, Doha, Qatar; 5 Department for Continuing Education, University of Oxford, Oxford, UK; 6 School of Science and Technology, University of New England, Armidale, NSW Australia; 7 Geelong Cats Football Club, Geelong, VIC Australia

**Keywords:** Team effectiveness, Team performance, Leadership, Teamwork, Organisation

## Abstract

**Background:**

The primary aim of our systematic scoping review was to explore the factors influencing team function and performance across various industries and discuss findings in the context of the high-performance sport support team setting. These outcomes may also be used to inform future research into high-performance teamwork in sport.

**Methods:**

A systematic scoping review of literature published in English since 2000 reporting team-based performance outcomes and included a performance metric that was ‘team outcome based’ was conducted using search of the Academic Search Ultimate, Medline, Business Source Ultimate, APA PsycInfo, CINAHL, SPORTDiscus, and Military database (ProQuest) using the terms: ‘team’, ‘function’ OR ‘dysfunction’, ‘Perform*’ OR ‘outcome’.

**Results:**

Application of the search strategy identified a total of 11,735 articles for title and abstract review. Seventy-three articles were selected for full-text assessment with the aim to extract data for either quantitative or qualitative analysis. Forty-six of the 73 articles met our inclusion criteria; 27 articles were excluded as they did not report a performance metric. Eleven studies explored leadership roles and styles on team performance, three studies associated performance feedback to team performance, and 12 studies explored the relationship between supportive behaviour and performance. Team orientation and adaptability as key figures of team performance outcomes were explored in 20 studies.

**Conclusions:**

Our findings identified 4 key variables that were associated with team function and performance across a variety of industries; (i) leadership styles, (ii) supportive team behaviour, (iii) communication, and (iv) performance feedback. High-performance teams wishing to improve performance should examine these factors within their team and its environment. It is widely acknowledged that the dynamics of team function is important for outcomes in high-performance sport, yet there is little evidence to provide guidance. This inequality between real-world need and the available evidence should be addressed in future research.

**Supplementary Information:**

The online version contains supplementary material available at 10.1186/s40798-021-00406-7.

## Key Points

- Across multiple sectors, four key variables were identified as important for teamwork, team function, team performance and team effectiveness; (i) leadership style (ii) supportive team behaviour (iii) communication, and (iv) performance feedback.
- Evidence obtained in this literature review was unable to illicit causal relationships between the four key variables important for high-performance sport support team function and individual athlete or playing team performance.
- Considering factors associated with teamwork, team function team performance and team effectiveness from other sectors provides leverage points for high-performance sport support teams to improve functions.

## Introduction

Each team has the potential to rise or fall based on the group of people who share the same passion and goals and are working together to achieve success [1]. This narrative is very common in elite sport, an environment that presents considerable health and performance challenges to the athlete and those charged with the responsibility of supporting them [2]. Considering that the success of athlete support teams is often measured by athletic performance outcomes [3], evidence supports the notion that contemporary athlete achievement can be strongly influenced by the function of the athlete support team [4, 5]. However, given the enormity of the performance and health challenges, elite sport teams may need further inputs beyond traditional structures of coaching staff and limited number of medical personnel to influence health and athletic performance outcomes [6]. Research exploring the dynamics of team function and team performance in an elite sporting environment is one under-appreciated area that can assist meeting this increasing challenge. The nature of team function is a complex phenomenon that is far from resolved [1].

**Fig. 1:**
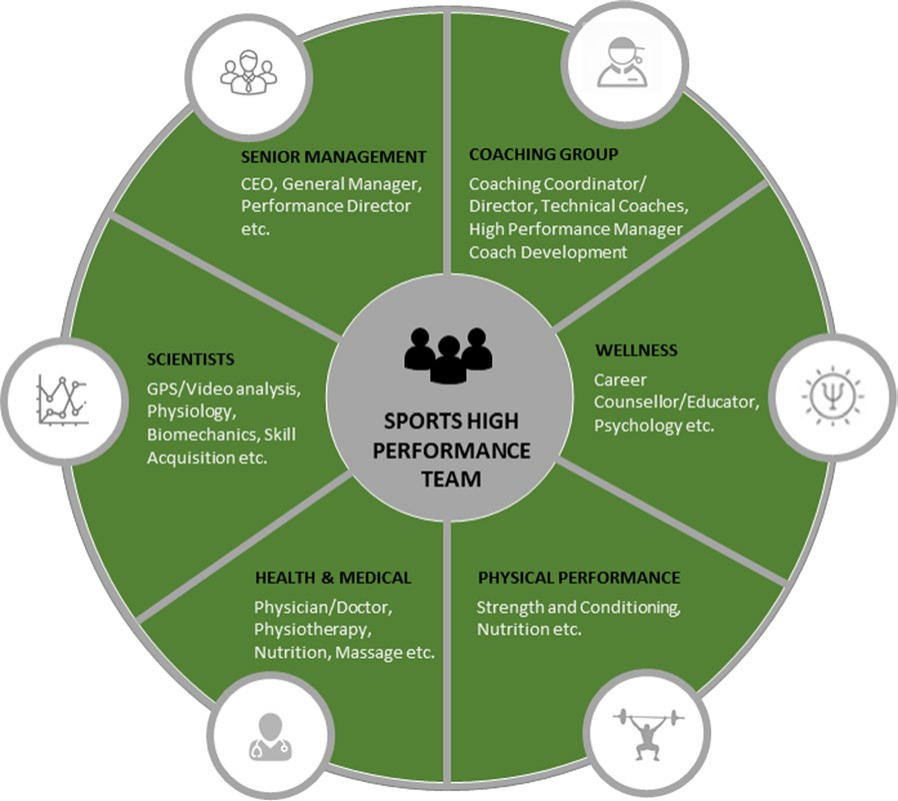
A model of the support team in high-performance sport

A ‘team’ can be defined as a group of individuals with specified roles and responsibilities interacting adaptively, interdependently, and dynamically towards a valued common outcome and who are together embedded in an encompassing organisational system, with boundaries and linkages to the broader system context and task environment [7]. Individuals within elite sport support teams include team/athlete coaches and the sports medicine and science team members who are constantly looking for ways to improve the performance and health of the athletes with whom they work [8]. Although varying in definition across sporting contexts, this team of individuals supporting the athlete form the high-performance team (HPT; see Fig. 1) [2, 9–11]. Teamwork refers to the behavioural processes that team members (e.g. members of a HPT) use to achieve work within the team (e.g. communication, collaboration, sharing of expertise), and team function refers to a group of people working towards a common objective. That is, the function of a team relates to the ability to coordinate and cooperatively interact with each other to facilitate task objectives through a shared understanding of the team’s resources (e.g., members’ knowledge, skills, and experiences), the team’s goals and objectives, and the constraints within the work environment [12–14]. Thus, teamwork is a component of team function [15, 16]. Team performance accounts for the cumulative outputs of the team’s actions, sometimes irrespective of how the team may have accomplished the task [7]. The effectiveness of a team, however, takes a holistic perspective in considering not only how the team performed, but also how the team interacted attempting to achieve a desired output (see Additional file 1) [15]. Thus, the performance of support teams in high-performance sport may not be simply reduced to the outcomes of the athletes or teams of athletes they support.

Teams that encourage and facilitate each other’s efforts in order to reach a common goal are influenced by issues of leadership [17], supportive team behaviour [18], organisational environment [19] and adaptability [20]. Teams educated about the mechanisms of teamwork (performance monitoring, adaptation, and facilitative leadership) have better performance outcomes [21], particularly when team members were able to anticipate each other’s behaviours and had better communication mechanisms. The addition of coordinating mechanisms such as supportive team behaviour, team communication and orientation are necessary facilitators of teamwork for a team to be successful [7, 22]. Furthermore, the high-performance sporting environment presents challenges for individuals to function effectively as a team [23]. Despite increased interest in the teamwork construct [24, 25], there are multiple and divergent conceptualisations of teamwork. There is a limited perspective in the present literature regarding the teamwork–team performance relationship [26]. To the authors’ knowledge, little work has described what the inputs and processes of teamwork are, nor described methodologies to measure the various influences and determine their role in assessing teamwork relative to performance in high-performance sport.

Challenges within HPTs in the elite sport setting arise because of factors such as organisational climate, professional conflict, power and influence challenges coupled with employment insecurities [19]. Additionally, high risk to reward scenarios, the demand to have a competitive advantage, and the emphasis on winning, have fractured the modern sports culture resulting in disparity and separation of athlete support staff and coaching staff within the same team [2, 27, 28]. Effective team function underpins the achievement of desired outcomes of collaborative work [12]. Consequently, suboptimal teamwork has at times catastrophic results for outcomes of such work [29]. While high-performance teams in elite sport have benefited from considerable scientific advances in physical preparation, participation and recovery practices, elite sport in this instance has not benefited from the science of teamwork effectiveness [30]. The primary aim of our systematic scoping review was to explore the factors influencing team function and performance across various industries and discuss findings in the context of the high-performance sport support team setting. These outcomes may also be used to inform future research into high-performance teamwork in sport.

## Methods

We adopted the Preferred Reporting Items for Systematic Reviews and Meta-analysis extension (PRISMA-ScR) guidelines [31] to identify a primary set of articles for data extraction and review. The 5-step process as described by Arksey and O’Malley [32] with enhancements as described by Levac et al. [33] was utilised: Identify the research question, identify relevant studies, study selection, chart the data, and collate, summarise, and report the results. In the final step, the review process was supplemented by application of thematic analysis methods [34] to categorise each article within the themes that emerged from relevant literature on team effectiveness models [7, 35–37]. The PRISMA extension for scoping reviews (PRISMA-ScR) checklist was used to ensure complete and transparent reporting [31].

### Identification of Relevant Studies

The article inclusion criteria were; full text, empirical studies published in English, between 2000 and November 2021, and reported objective team-based performance outcomes and included a performance metric that was ‘team outcome based’, e.g., team effectiveness, cohesiveness, efficiency, reflexivity and potency. We chose to explore only articles with an objective performance based outcome to limit theoretical/speculative content. Articles were excluded under the following criteria: the study had no defined metric of performance outcomes, was a literature review or was an opinion piece.

A search of the Academic Search Ultimate, Medline, Business Source Ultimate, APA PsycInfo, CINAHL, SPORTDiscus, and Military database (ProQuest) was conducted in October 2021 using the terms: ‘team’, ‘function’ OR ‘dysfunction’, ‘Perform*’ OR ‘outcome’. All records retrieved by the search query were imported into Endnote X9 (Thompson Reuters, Carlsbad, CA, USA) and duplicates removed.

### Final Study Selection

Two authors (BS, BGS) independently reviewed titles and abstracts for potential eligibility. For the potentially eligible records, the full-text articles were thereafter retrieved and assessed according to the inclusion and exclusion criteria. The reference lists of the resulting articles were searched by the lead author (BS) for inclusion of additional articles. Any discrepancies were discussed by the reviewers (BS, BGS). No conflicts were identified. The review of full-text articles revealed that those articles that reported a performance metric provided sufficient content data for a continued analysis.

### Collating the Results

Analysis of the methodological and conceptual features of extracted data was thereafter performed by the lead author (BS) to summarise and collate the content of the articles and was subsequently confirmed by a co-author (BGS). Analysis of eligible papers involved describing the type of study which was performed, the occupational domain the study was conducted, where it was conducted, participant characteristics, study aims, performance metric and the category of teamwork. With regards to the conceptual analysis, we focused on examining common and emerging themes among definitions of team performance and their operationalisation (e.g., leadership, team orientation) as well as primary research findings as they pertained to team performance. A critical appraisal was not conducted on our findings as the aim of this review is to identify and map the available evidence [32].

**Fig. 2:**
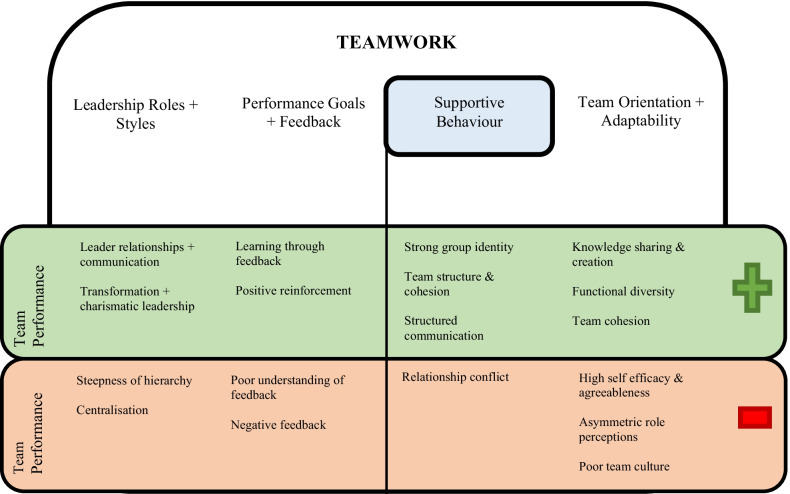
Concept chart illustrating the characteristics of teamwork and how they are associated with team performance outcomes

The operationalisation categories followed the key themes of teamwork that emerged from the literature on team effectiveness models [7, 12]. *Team leadership roles and styles*; the ability to direct and coordinate the activities of other team members, assess team performance, assign tasks, develop team knowledge, skills, and abilities, *performance goals and feedback*; the ability to develop common understandings of the team environment and apply appropriate task strategies to accurately monitor teammate performance, *team orientation and adaptability*; the ability to adjust strategies based on information gathered from the environment through the use of supportive team behaviour and reallocation of intrateam resources, *supportive team behaviour*; the ability to anticipate other team members’ needs through accurate knowledge about their responsibilities (Fig. 2).

## Results

### Literature Search

**Fig. 3:**
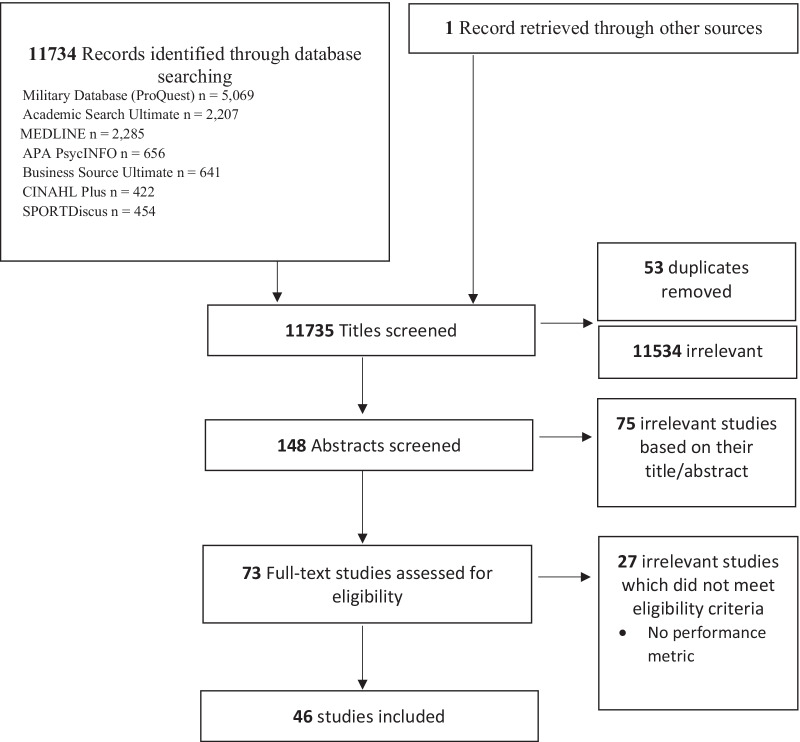
PRISMA flow chart showing the process for including studies

The initial literature search identified a total of 11,734 articles for title and abstract review, and one article was retrieved from another source. Seventy-three articles were selected for full-text assessment with the aim to extract data for either quantitative or qualitative analysis. Forty-six of the 73 articles met our inclusion criteria; 27 articles were excluded as they did not report a performance metric. The article selection process is seen in Fig. 3.

### Study Characteristics

**Fig. 4:**
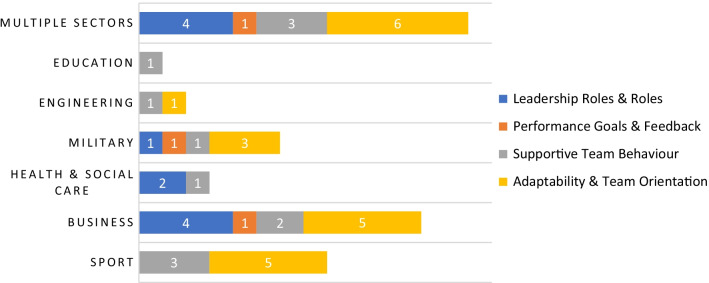
Trend of the number of articles found between the various workplace domains

**Fig. 5:**
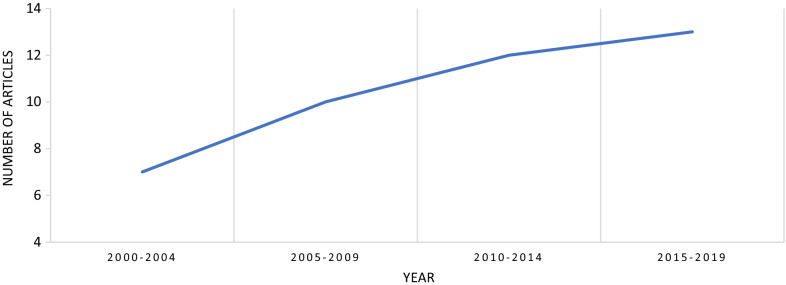
Trend of the number of articles found over the two-decade period 2000–2020. Note, articles from 2020 to 2021 are not included in this graph so that we can better demonstrate increased article production over time using evenly distributed time brackets

The 46 papers identified from the search process were published across a twenty-year period (2000–October 2021) (Figs. 4, 5). Team performance outcomes were examined within business (*n* = 12), sport (*n* = 8), military (*n* = 6), health and social care (*n* = 3), engineering (*n* = 2), education (*n* = 1) or across multiple sectors (*n* = 14) (Fig. 4). In terms of geographical location, the studies were conducted across: North America 61% (USA, *n* = 26; Canada, *n* = 2), Europe 28% (UK, *n* = 4; Netherlands, *n* = 3; Spain, *n* = 2; Germany, *n* = 1; Italy, *n* = 1; Portugal, *n* = 1; Europe, unknown = 1), Asia Pacific 9% (South Korea, *n* = 1; Pakistan, *n* = 1; India, *n* = 1; Australia, *n* = 1), Africa 2% (Tunisia, *n* = 1). There was a positive trend of the number of articles produced over the 2-decade period, 2000–2004 (*n* = 7), 2005–2009 (*n* = 10), 2010–2014 (*n* = 12) and 2015–2019 (*n* = 13) (Fig. 5).

Studies utilised mixed methods approaches (*n* = 17) (i.e., questionnaires combined with archival data from financial reports and published articles), cross-sectional surveys (*n* = 12), experimental interventions designed to evaluate team performance among participants (*n* = 8), and interview-based approaches (*n* = 1). Other designs included archival analysis (*n* = 5) and laboratory-based experiments (*n* = 3).

### Outcomes

#### *Team Leadership Roles *and* Styles*

Eleven studies explored leadership influences on team performance (Table 1) [38–48]. The role of the team leader is described as pivotal for effective team function, as leaders have responsibility for team members and the direction of all team activity and processes [45, 49]. Leaders who displayed higher cognitive ability, conscientiousness and charisma were better able to mediate their teams to enhance team performance outcomes [40, 41, 43, 44, 46]. Charismatic and transformational leaders positively change the values and priorities of team members and motivate them to perform beyond their expectations [39, 41]. Leaders within a centralised structure where the decision-making authority is concentrated at the top, and all other lower levels follow the directions coming from the top of the organisation structure, have negative effects on conflict and performance. This leadership model also affects relationships between team members [38, 42, 46]. Our literature search revealed that teams will perform better when team leaders are highly involved in the team’s communication and workflow networks [42, 45]. Specifically, in diverse work groups, the nature of interpersonal interactions was found to be an important determinant of group member performance and group effectiveness. For example, referring to gender diversity, one of the studies retrieved argued that a diverse group with low leader-member relationships (i.e. where relationships between team leaders and team members were poor) will not perform highly regardless of how well the leader differentiates role assignments because of insufficient attention to relationships [46]. Leaders act as influential role models, wherein their self-regulatory behaviours directly shape task-related team processes, which was shown to positively influence team performance [44].

**Table 1:** Characteristics of studies within leadership roles and styles

Author/Year	Aim	Study population	Findings
Business			
D'Innocenzo et al. (2021) [47]	To test (i) whether shared leadership and performance were related over time, (ii) the relative magnitude of those relationships, (iii) whether a shared leadership intervention changes those relationships	205 Members of 53 student teams in business	Shared leadership to performance relationship was positively related and grew stronger with intervention
Doghri et al. (2021) [48]	Analyse the influence of empowerment leadership and its mechanism of effect	250 Start up businesses	Shared leadership and knowledge sharing had a positive relationship on performances
Cicero and Pierro (2007) [39]	Analyse leadership and work outcomes as they are associated to social identification processes	200 Italian public and private sector employees	Positive association between charismatic leadership and employees’ work-group identification; work-group identification is also associated with their job involvement, job satisfaction, performance, and turnover intention
Payne et al. (2009) [45]	Identify five attributes of high-performing teams—knowledge, information, power, incentives and opportunity/time—and how they influence corporate financial performance	210 Fortune 1000 companies	Team effectiveness attributes are associated with higher levels of board effectiveness as rated by the board directors, and that board effectiveness is significantly related to corporate financial performance
Military			
Stewart and Johnson (2009) [46]	Test a moderator of the association between diversity and work group performance: leader–member exchange (LMX)	224 High-ranking officers	Leader-member exchange interacted with work group gender diversity, such that in more gender diverse groups leader–member exchange differentiation was positively associated with work group performance when aggregate leader–member exchange was high (above the median)
Health and social care			
Kane and Borgatti (2011) [42]	Examine if groups will perform better if their more proficient members are highly central in the group’s communication and workflows network	468 Employees (32 teams)	Centrality–Information System proficiency alignment is significantly and positively related to performance across multiple systems examined individually and with the portfolio of systems examined
Kickul and Neuman (2000) [43]	Investigate the individual differences in emergent leadership behaviours and their relationships to teamwork processes and outcomes	320 Psychology students	Openness to experience and cognitive ability were predictive of emergent leadership behaviours. Conscientiousness and cognitive ability were associated with team performance
Multiple sectors			
Bunderson, van der Vegt et al. (2016) [38]	Explore whether hierarchy can promote group performance and member satisfaction	75 Teams	Acyclicity in influence relations reduces conflict and thereby enhances both group performance and member satisfaction, centralisation and steepness have negative effects on conflict, performance, and satisfaction, particularly in groups that perform complex tasks
DeChurch and Marks (2006) [40]	Leader strategising and coordinating effects on functional leadership, inter team coordination, and multiteam systems performance were examined	384 Undergraduate students	Functional leadership mediated the effects of both types of training on inter team coordination and inter team coordination fully mediated the effect of multiteam system leadership on multiteam system performance
Lyubovnikova et al. (2017) [44]	Examine how authentic leadership influences team performance via the mediating mechanism of team reflexivity	53 Teams	Self-regulatory behaviours inherent in the process of authentic leadership served to collectively shape team behaviour, manifesting in the process of team reflexivity, which, in turn, positively predicted team performance
Han et al. (2018) [41]	Investigate how an organisation's high-performance work system affects team managers' transformational leadership	179 Teams in 44 organisations	Multilevel, moderated mediation effect with the indirect effect of high-performance work system on team performance via transformational leadership varies significantly as a function of adaptation and efficiency orientations

#### Performance Goals and Feedback

Three studies associated performance monitoring to team performance (Table 2) [50–52]. They explored the use of negative feedback and positive reinforcement as modalities for performance feedback and argued this can help to build the team, the culture, and the capacity for quality improvement [50–52]. They showed, learning through performance feedback provides team members with the opportunity to learn how to work collaboratively [52], having the potential to (1) shape team culture or attitudes, (2) establish common team goals, and (3) improved understanding of performance standards [51]. However, in one study, it was noted that the effect of team performance feedback on intentions to improve performance was hindered by a poor understanding of how the team could use the feedback and how the feedback was perceived [51].

**Table 2:** Characteristics of studies within performance goals and feedback

Author/Year	Aim	Study population	Findings
*Business*			
Bachrach et al. (2001) [50]	Examine the possibility that feedback regarding team performance may influence team members' reports of organisational citizenship behaviours	95 Teams of business students	Organisational citizenship behaviour (helping behaviour and civic virtue) in work groups may be a function of the nature of the performance feedback that group members receive; negative feedback plays a more critical in this attributional process
*Health and social care*			
Kotecha et al. (2015) [52]	Explore the influence of the learning collaborative program on team functioning in participating primary healthcare teams	10 PHC teams	The learning collaborative program provided opportunities for participants to learn how to work collaboratively, and participation in the learning collaborative program appeared to enhance team functioning
*Multiple sectors*			
Johnston et al. (2011) [51]	Explore the acceptability and impact of feedback of team performance data to primary care interdisciplinary teams	7 Interdisciplinary teams	Existing performance indicators do not equally reflect the role of different disciplines within an interdisciplinary team

#### Supportive Team Behaviour

Eleven studies [26, 53–62] explored how the relationship between supportive team behaviour, the ability to anticipate other team members’ needs through accurate knowledge about their roles and responsibilities [7], and team performance, complement each other (Table 3). Teams with strong group identity, communication and structural cohesion mitigated the adverse consequences of team conflict and collective team failure [53, 56, 60, 61, 63], Relationship conflict within teams has negative consequences on task performance [57, 59]. Task conflict has positive impacts on team performance in teams exhibiting high levels of openness and emotional stability [54, 55, 57]. Members within teams that engage in more cooperative behaviours become more efficient, effective, and viable [55, 56, 60, 61]. Supportive team behaviour has additional positive effects on team performance when in combination with performance monitoring [26].

**Table 3:** Characteristics of studies within supportive team behaviour

Author/Year	Aim	Study population	Findings
*Business*			
Bradley et al. (2013) [55]	Investigate whether personality compositions influence the effect of task conflict on team performance	561 Students	Task conflict had a positive impact on performance in teams with high levels of openness or emotional stability
Porter et al. (2010) [26]	Examine boundary conditions for the positive effects of two aspects of teamwork (backing up behaviour and performance monitoring) on team performance	276 Undergraduate business students (69 teams)	Backing up behaviour had positive effects on team performance when combined with performance monitoring
*Engineering*			
Shaukat et al. (2017) [59]	Offer insights regarding the consequences of relationship conflict among employees in terms of their task performance, contextual performance and turnover intentions	306 Telecom engineers	Relationship conflict is negatively related to task performance, contextual performance and turnover intentions
*Sport*			
You (2020) [62]	Analyse the difference in cultural functions between high- and low-performance university soccer teams	316 Korean University Soccer Players	Higher performing teams were good at adapting to changes in their environment, and had coherent and aligned goals
Verma et al. (2012) [60]	Assess the role of different parameters of team cohesiveness (Group-Task and Group-Social) among the high and low performing teams	208 Male elite volleyball players	Group cohesion parameters were significantly higher among high-performance volleyball players in comparison with low-performance players
Warner et al. (2012) [61]	Employs social network analysis as a tool to explore a case study of the structural cohesiveness of two women’s collegiate basketball teams	47 Team members	High performing teams showed improved structural cohesion in the efficacy network and highlighted the movement of key players in the different networks (friendship, trust, advice, and efficacy) over time
*Health and social care*			
Jehn et al. (2015) [57]	Examine the effects of asymmetric perceptions of task conflict on the anticipated relationship with the partner, as well as subjective and objective performance	84 University students (25 men and 59 women)	When individuals realise that they have asymmetric task conflict perceptions, they have lower expectations about having a positive relationship with their partner and perform worse
*Education*			
Reimer (2001) [58]	Explore the effect of performance attributions on group achievement	80 Senior High school students	In situations in which a conflict arises among group members group, performance is determined by the individual’s problem-solving strategies and also by the extent to which group members consider their partners’ perspective
*Multiple sectors*			
Bachrach et al. (2006) [53]	Examine whether task interdependence moderates the relationship between the helping form of organisational citizenship behaviour (OCB) and group performance	62 Teams	The relationship between helping and group performance depends on the level of task interdependence required of group members
Jackson (2011) [56]	Investigate if group failure on a task was expected to adversely affect cooperative responses to a subsequent social dilemma	48 Four-person teams	A strong group identity mitigated the adverse consequences of collective failure
Bang and Park (2015) [54]	Examine the relationship between task conflict and team performance	5,579 Employees (153 teams)	Task conflict positively predicted actual team performance when job demand was high, whereas it had a negative effect when job demand was low

#### Team Orientation, Organisational Context and Adaptability

Team orientation, organisational context and adaptability as key features of team performance outcomes were explored in twenty-one studies (Table 4) [38, 63–82]. Team orientation describes how members in teams learn, store, use, and coordinate their knowledge to accomplish team and organisational goals [76]. Team communication and cohesion were found to be key to collaborative work within teams to enhance team performance [63, 68, 69, 75, 80]. Functional diversity within teams had varying implications for team processes and performance depending on how this was utilised [83]. Specifically, intrapersonal functional diversity—where each member’s experience is distributed over many functional domains (operations, logistics, leadership), rather than focused on one specific functional area—was positively associated with information sharing and collective group performance [68, 83]. The right processes and team culture in an organisation promote team commitment [37]. Organisational context influences team effectiveness, both directly and by determining the initial conditions that promote effective team functioning [84].

**Table 4:** Characteristics of studies within adaptability and team orientation

Author**/**Year	Aim	Study population	Findings
*Business*			
Bakker et al. (2008) [67]	Examine how job characteristics and burnout (exhaustion and cynicism) contribute to explaining variance in objective team performance	176 Employees	Work conditions influence performance particularly through the attitudinal component of burnout
Bunderson and Sutcliffe (2002) [83]	Examined the process and performance effects of dominant function diversity and intrapersonal functional diversity	438 Individuals	Different forms of functional diversity can have very different implications for team process and performance; intrapersonal functional diversity matters for team effectiveness
Kong et al. (2015) [73]	Examine the view of team agreeableness as a moderator for the relationship between team member satisfaction and team performance	230 Senior-level professionals	When team agreeableness was low, team member satisfaction was positively related to team performance; no significance was found when team agreeableness was high
Kurtulus (2011) [74]	Explore the consequences of grouping workers into diverse divisions on the performance of employees	9248 Workers	Relationships between performance and the various measures of dissimilarity vary by occupational area and division size
Lewis (2004) [76]	How transactive memory systems emerge and develop to affect the performance of knowledge-worker teams	64 Consulting teams (*n* = 261)	Transactive memory systems were positively related to team viability and team performance, suggesting that developing a transactive memory system is critical to the effectiveness of knowledge-worker teams
*Engineering*			
Hirst et al. (2018) [72]	Examine domain-specific evidence that when individual self-efficacy is high, team climate has diminishing performance and creative benefits	317 Engineers	Team level and individual-level influences that by themselves are positive antecedents of performance and creativity in combination yield diminishing return
*Health and social care*			
Marques-Quinteiro et al. (2020) [82]	Team adaptability and cohesion affects absenteeism from work for firefighters	27 Firefighter teams	Absenteeism was less related to team cohesion when compared to workload
*Sport*			
Arnold et al. (2016) [65]	Examine if the frequency, intensity, and duration of the organisational stressors that sport performers encounter vary as a function of performance level	1277 Sport performers	Significant differences were found between males and females, between team and individual-based performers, and between performers competing at different levels
Buran et al. (2019) [69]	Establish if consistent concepts exist among sports medicine professionals working within elite cricket when developing a multidisciplinary performance team	6 Sports medicine professionals	Communication is key to members collaborative work within a multidisciplinary team, along with innovation and strong structural, philosophical, strategical and governance policies to enhance team performance
Carmichael and Thomas (2000) [70]	To estimate a production function for English Premiership football	20 Teams in premiership football	Player skills of accurate and effective shooting and passing, together with good defensive skills have a positive effect on team outcomes
Leo et al. (2013) [75]	Define different profiles of cohesion and perceived efficacy in soccer players and measure their differences in performance	235 Soccer players in the U18 +	Soccer players with higher cohesion and collective efficacy levels belonged to teams that completed the season at the top-level classification
Sánchez et al. (2007) [78]	Assess empirically the relative importance of the key factors determining a basketball team performance	18 Teams over 34 league days (2 seasons)	There is a substantial difference between the impact of each play characteristic on a team’s winning probability and that probability varies as the quality/ quantity of the input’s changes, albeit not proportionally
*Military*			
Aaberg et al. (2009) [64]	Investigate utilisation of a human performance model to explore and analyse a training organisation	Military organisation	The systemic and systematic practices of the human performance model are applicable to military organisations
Arthur Jr et al. (2012) [66]	Develop an effective method to identify team-based tasks and jobs and how they relate to team performance	140 F-16 Pilots	Teams that accurately perceived the level of interdependency performed better
Wright and Kaber (2005) [81]	Investigate effects of automation as applied to different stages of information processing on team performance in complex decision-making tasks	40 Teams	An increase in automation of information analysis resulted in higher team coordination ratings
*Multiple sectors*			
Brodbeck and Greitemeyer (2000) [68]	To compare individual training conditions with mixed group and individual training conditions on subsequent nominal and collective group performance	132 Students	Collective group performance improves as a function of group experience; nominal group performance improves as a function of improved individual resources for performing the task individually
Butchibabu et al. (2016) [63]	Evaluate the frequency and methods of communications used as a function of task structure	13 Teams	Teams in which members proactively communicated information about their next goal to teammates exhibited improved team performance
Firth et al. (2015) [71]	Study the effects of frame-of-reference training on multiteam system coordination and performance	249 Multiteam systems	Frame-of-reference training had a positive effect on team performance
Song (2008) [79]	To assess the impacts of knowledge creation process on organisational performance improvement	481 From Korean organisations	Knowledge creation practices could account for 40% of organisational performance
Sousa Pinto and Lourenço (2014) [80]	Analyse the relationship between the internal functioning of teams and their team task performance, as well as the moderating role of task interdependence in that relationship	72 Work teams (408 members)	The dimensions of the internal functioning of work teams are positively related to team performance
Mell et al. (2014) [77]	Compare teams in which metaknowledge is concentrated within one central member with teams in which metaknowledge is distributed evenly among the members	122 Individuals	Transactive memory systems allow teams to capitalise on the diversity of the knowledge held by their members by supporting coordination and integration of knowledge

A relationship exists between team performance and measures of demographic similarity; described as the team’s agreeableness, self-efficacy and creativity [73, 75, 83], and demographic diversity of age and sex [65, 74], In individuals low on self-efficacy and agreeableness, team climates encouraging exploitation and exploration respectively deliver increasing performance and creative benefits. When team encouragement for exploitation—treating someone unfairly in order to benefit from their work—increases, the returns on such encouragement diminish, and individuals with high levels of self-efficacy and agreeableness show less additional performance and creative returns [72, 73]. Age, job tenure and performance dissimilarity are also associated with lower team performance as broader contextual factors in the social world are potential obstacles to effective team functioning [65, 74].

## Discussion

This systematic scoping review identified four key variables that were associated with team function and performance across a variety of industries; (i) leadership styles [17], (ii) supportive team behaviour [18], (iii) communication, and (iv) performance feedback [20]. High-performance teams may wish to consider prioritising these variables to improve health and performance outcomes. However, this should be done with caution given limited evidence was identified in sport relative to these factors. Team function and performance in the context of support teams in high-performance sport may be better enhanced if we first work towards understanding the behaviour of those four key variables relative to each other in the broader sports team [85].

### Leadership Styles Influence Team Cohesion and Performance

In sport, leadership behaviour is not just important for individual players; it is important for the team as a whole as it establishes an interpersonal environment characterised by support, respect, trust and appreciation of staff and players [86], which ultimately have a positive influence on team cohesion and performance [86]. Leadership styles that promote back up behaviour were suggested to enhance team cohesion. Highly cohesive teams worked together more efficiently and, consequently, performed better than less cohesive teams [39]. It is well established that leadership serves as a critical input for influencing group processes and output, and that leaders can shape team members’ attitudes, beliefs, and values [44]. Sports psychology research supports the view that leadership behaviours are associated with higher levels of motivation and performance [87–89], increased well-being [90], and increased task/team cohesion [87]. A study of leadership styles of football coaches indicated that leadership behaviours that communicated a clear and positive vision of the future appeared to reduce the risk of severe injuries by 29%-40% [86]. This is in line with the idea that transformational leaders develop an image of the future of their organisation and communicate that vision to their subordinates. In contrast, leadership that does not promote supporting behaviour and adaptability might risk insufficient collaboration within the team, poor decision-making and high stress. This is likely to lead to the team underperforming [11].

Our findings demonstrate that charismatic leadership has positive effects on team performance [39]. This is contrary to the evidence supporting this style of leadership within the sport setting. In a recent study in the sport of football [86], no correlation was found between charismatic leadership and injury rates or players’ availability. It is incumbent on the leader to establish positive rapport across the team as this is an important determinant of team performance and effectiveness [46].

### Team Communication and Feedback Influence How a Team May Function

Open communication and feedback about both strengths and weaknesses were identified as a characteristic of well-performing teams, and poor communication was a marker of dysfunctional relationships [91]. When teams of multidisciplinary practitioners adopt this teamwork approach, they have been described as an ‘interdisciplinary team’, differentiated by their integration of knowledge and collaborative behaviours beyond that seen in ‘multidisciplinary teams’, where individuals work towards their own goals with limited interaction [84, 92]. This may be explained by the mechanism through which teams collectively encode, store, and retrieve knowledge; described as transactive memory systems (TMS). TMS facilitates team shared knowledge and communication by developing a structure and organisation [64, 67, 69, 77, 79–81], and supporting the development, integration and change of knowledge and its content [79].

Communication is considered an important mediator of performance in team sports [93]. This notion is supported by work which highlights the importance of distributed decision-making in groups of people [94], and in fact, a recent study in the sport of football concluded that the quality of communication within a team was associated with both injury rates and player availability [91]. Teams with high internal communication quality had lower injury rates and higher player availability than teams with low communication quality [91]. Low communication quality between the head coach and the medical team was significantly associated with the injury rate; such teams had a 6%–7% lower player availability at training and matches and a 50% higher injury burden, compared with teams with moderate or high communication quality [91]. High quality communication between individuals in different roles is likely to promote good collaborations and facilitate the benefits derived from multiple perspectives in informed decisions, for instance, return to play decision or major decisions regarding the well-being of players [2, 91].

Low-quality communication is likely to increase the risk of misunderstandings and promote one-sided decision-making and high stress, which in the long run might contribute to the risk of injuries [11, 91]. Without effective communication and feedback, it is difficult to modify individual training plans (e.g. training load and other environment considerations like training surface) according to athlete age, position and medical history. Good communication, management and training restrictions can assist players to continue playing and performing throughout the season without exacerbating the injury [91]. The tendency to weight negative information more heavily than positive information during feedback processes could help account for the asymmetrical effects that negative (as opposed to positive) feedback has on group members' implicit performance [50]. Feedback strongly influences emotional reactions, which in turn affect employees' attitudes and role behaviours. Therefore, leaders may be better off framing their feedback to subordinates in a positive rather than a negative manner as this comes with increased employee commitment and organisational citizenship behaviour [95]. Considering teamwork factors that have been demonstrated to shape outcomes of teamwork in organisations outside of sport provides leverage points for teams to improve team function [25].

### Team Culture May Mitigate Against Consequences of Team Conflict

Team culture—a shared set of values that inform a group’s behaviour—is considered one of the most prominent contributors to the success of a sporting organisation [96, 97]. Teams with strong team culture mitigate the adverse consequences of team conflict and collective team failure [53, 56, 60, 61, 63] as it facilitates supportive behaviour and accountability by having clear purpose, well-defined roles and organisational policies [10, 98]. In the sport setting, there are established hierarchies based around teamwork [2]. The organisational culture and climate of elite sport have been described as ‘rife’ with culturally-driven challenges that include interdepartmental communication problems, coach-athlete conflict, interference from owners, negative reporting in the media and staff being required to continually justify how their input impacts performance [23]. Sports teams that foster acceptance of group goals, promote communication and positive conflict had a positive relationship with team cohesion [99]. Teams who are able to address conflict directly are better able to develop an open constructive atmosphere and forge a stronger team identity [100]. However, HPT may exhibit high levels of team conflict, particularly within high pressure environments like that in elite sport [23] which can interfere with effective team performance [101]. When team members’ perceptions of their individual role within the team are in alignment with how other team members perceive their roles, HPT can avoid high levels of team conflict and exhibit better team performance [101].

### Bias, Limitations and Future Research

Within our systematic scoping review, we identified commonly interchangeable use of terminology which makes pooling and summarising the results across industries and domains difficult. The studies identified displayed a publication bias towards cross-sectional studies. Such study designs are unable to assess the dynamic nature of working in teams. Teams are complex, dynamic systems that ‘adapt’ to new knowledge, relationships, external events and environment constraints among many other potential inputs. It is therefore important to carefully consider optimal study designs when examining team behaviours and their consequences [7] through certain study designs. Future research to agree on a taxonomy of definitions will enable research in this area to be applied to a sporting context and compared across investigations. An expected limitation of this review was the lack of existing research that satisfied the search criteria for data extraction. To minimise this limitation, we searched a common array of academic research databases leading to a sensitive search strategy which identified many false positives based on the inclusion criteria. No studies identified in this systematic scoping review investigated causal relationships. Future research investigating whether certain inputs or process improve team function may benefit from utilising causal inference methodology.

We concede this review has explored the effect of support team-teamwork/team effectiveness/team function on injury incidence and availability of athletes; however, its effect on athlete or playing team sporting performance has not been commensurately discussed. To the knowledge of the researchers, no evidence linking support team-team work to individual or playing team sporting performance exists. If we are to consider, however, increased athlete availability increases training opportunity, and that the people in the broader team environment can affect competition performance in athletes [102–104], it is reasonable to assume support team-teamwork/team effectiveness/team function affects athlete or playing team sporting performance similarly to how it affects athlete injury incidence and availability.

## Conclusion

Across various sectors, we identified that improved team function and performance are associated with leadership, supportive team behaviour, communication, and performance feedback. In the context of complex sporting organisations where leaders must respond to multiple stakeholders and meet performance goals across multiple dimensions of effectiveness, addressing the reported challenges and considering the importance of organisational commitment to team development can help ensure that team objectives are effectively designed, delivered, and sustained. While the evidence obtained in this literature review was unable to elicit causal relationships between these factors and enhanced sport performance, it provides a point at which high-performance sport support teams can commence their investigation and interventions to improve team function and performance. This review will pave the way for future research; however, no agreement currently exists on terminology and definitions for performance outcomes to support performance analyses of teamwork and to establish if a performance support team that works effectively will enable better health and performance outcomes for their athletes/sport team. It is widely acknowledged that the dynamics of team function is important for outcomes in high-performance sport, yet there is a dearth of evidence to provide guidance in the high-performance sport context; hence, we have explored team work in alternate sectors. This inequality between real-world need and the available evidence should shape future research to work towards examining team effectiveness related to achieving both health and performance outcomes in elite sport.

## Supplementary Information

**Additional file 1.** Notes.

## Data Availability

All relevant data are included within this article.

## Abbreviations

HPT: High-performance team
TMS: Transactive memory systems
