# Supplementary material for: Factors Influencing Team Performance: What Can Support Teams in High-Performance Sport Learn from Other Industries? A Systematic Scoping Review
Source: Sports Med Open. 2022 Feb 22;8:25. doi: 10.1186/s40798-021-00406-7 (PMC8864029; doi:10.1186/s40798-021-00406-7)
Supplement: Supplementary file 1 — Additional file 1. Notes. [file 40798_2021_406_MOESM1_ESM.docx]

**Notes**

| **Term** | **Definition** |
| --- | --- |
| *Teamwork* | Behavioural processes (e.g. communication, collaboration, sharing of expertise) that practitioners use to accomplish independent or interdependent work and/or the affective, cognitive and motivation states that emerge during that work (e.g. cohesion).[15] |
| *Team effectiveness* | The capacity of the team to achieve goals/objectives and or expectations set internally or by external stakeholders over a given period.[15] |
| *Team function* | A group of people with a common functional expertise working toward shared objectives.[15] |
| *Multidisciplinary team* | Teams of professionals from different disciplines who work towards shared goals and within a team structure.[62] |
| *Interdisciplinary team* | Teams of professionals from different disciplines who work towards their own goals within a team structure.[95] |
| *High-Performance team (HPT)* | Individuals within elite sport teams include team / athlete coaches and the sports medicine and science team members who are constantly looking for ways to improve the performance and health of the athletes with whom they work. |
| *Team Culture* | A pattern of shared basic assumptions learned by a group as it solves its problems of external adaptation and internal integration’ [96] |
| *Team cohesion* | A group of individuals that is connected and driven to achieve a common goal.[7] |
| *Transformational leadership* | Leadership where a leader works with teams to identify needed change, creating a vision to guide the change through inspiration, and executing the change in tandem with committed members of a group. [45] |
| *Charismatic leadership* | A leader who uses his or her communication skills, persuasiveness, and charm to influence others.[39] |
| *Leader-member exchange* | A relationship-based approach to leadership that focuses on the two-way (dyadic) relationship between leaders and followers.[41] |
| *Organizational citizenship behaviour* | The positive and constructive employee actions and behaviors that aren't part of their formal job description.[47] |
| *Transactive memory systems* | A mechanism through which groups collectively encode, store, and retrieve knowledge. [75] |
| *Team leadership* | The ability to direct and coordinate the activities of other team members, assess team performance, assign tasks, develop team knowledge, skills, and abilities.[7] |
| *Mutual performance monitoring* | The ability to develop common understandings of the team environment and apply appropriate task strategies to accurately monitor teammate performance.[7] |
| *Adaptability* | The ability to adjust strategies based on information gathered from the environment through the use of backup behavior and reallocation of intrateam resources.[7] |
| *Backup / Supporting behavior* | The ability to anticipate other team members’ needs through accurate knowledge about their responsibilities.[7] |
| *Team orientation* | Propensity to take other’s behavior into account during group interaction and the belief in the importance of team goal’s over individual members’ goals.[7] |

**Note 1** Definitions of key terms
